# 1285. Systematic Literature Review of Real-world Experience with the 2-Drug Regimen Dolutegravir and Lamivudine in People with HIV Who Would Not Have Met Inclusion Criteria for the Phase 3 Clinical Program

**DOI:** 10.1093/ofid/ofac492.1116

**Published:** 2022-12-15

**Authors:** Jihad Slim, Douglas Ward, Stefan Schneider, Madhusudan Kabra, Gustavo Verdier, Clifford B Jones, Emilio Letang

**Affiliations:** Saint Michael’s Medical Center, Newark, NJ, USA, Newark, New Jersey; Dupont Circle Physicians Group, Washington, DC, USA, Washington, District of Columbia; Long Beach Education and Research Consultants, Long Beach, CA, USA, Long Beach, California; ViiV Healthcare, Brentford, UK, Brentford, England, United Kingdom; ViiV Healthcare, Montréal, QC, Canada, Pointe-Claire, Quebec, Canada; ViiV Healthcare, Brentford, UK, Brentford, England, United Kingdom; ViiV Healthcare, Madrid, Spain, Madrid, Madrid, Spain

## Abstract

**Background:**

In phase 3 randomized controlled trials (RCTs), dolutegravir/lamivudine (DTG/3TC) demonstrated durable efficacy in treatment-naive (GEMINI-1/-2) and virologically suppressed switch (TANGO, SALSA) participants. Eligibility criteria for these RCTs included no history of treatment failure or any major nucleoside reverse transcriptase inhibitor or integrase inhibitor–associated mutations, no hepatitis B virus (HBV) or need for hepatitis C virus (HCV) therapy, and viral load (VL) < 500,000 c/mL at screening (GEMINI) or < 50 c/mL for > 6 months (TANGO, SALSA). We analyzed real-world evidence (RWE) for DTG + 3TC use in people with HIV (PWH) with baseline characteristics not consistent with these inclusion criteria.

**Methods:**

We conducted a systematic literature review according to the Preferred Reporting Items for Systematic Reviews and Meta-analysis statement. RWE studies that reported on DTG + 3TC use in PWH were retrieved from Ovid MEDLINE^®^, Embase^®^, PubMed, Cochrane library, and relevant international conference proceedings from January 2013 to February 2022.

**Results:**

This review includes 122 publications from 103 RWE studies of 44 unique cohorts (N=8034; 42 cohorts outside the United States; Tables 1-2, Figure). In the 1 study that described outcomes in PWH with previous virologic failure (VF; N=194), probability of VF at 1 year was low (0.4% or 1.2%, depending on VF criteria). In cohorts with > 10 PWH with baseline resistance that reported outcomes (mostly M184V/I; 4 cohorts, N=211), VF was low (ranging from 0%-5.4% at ∼1 year), and the difference in VF between those with or without M184V/I was not significant in 3 of 4 cohorts. A treatment-emergent resistance mutation (M41L, not selected by DTG or 3TC) was observed in 1 PWH with evidence of baseline resistance. None of the 35 PWH with HBV experienced VF, and 89% (16/18) of treatment-naive PWH with baseline VL > 500,000 c/mL achieved virologic suppression at Week 24. There were no studies describing effectiveness outcomes in PWH with HCV who were receiving DTG/3TC.

**Figure:**
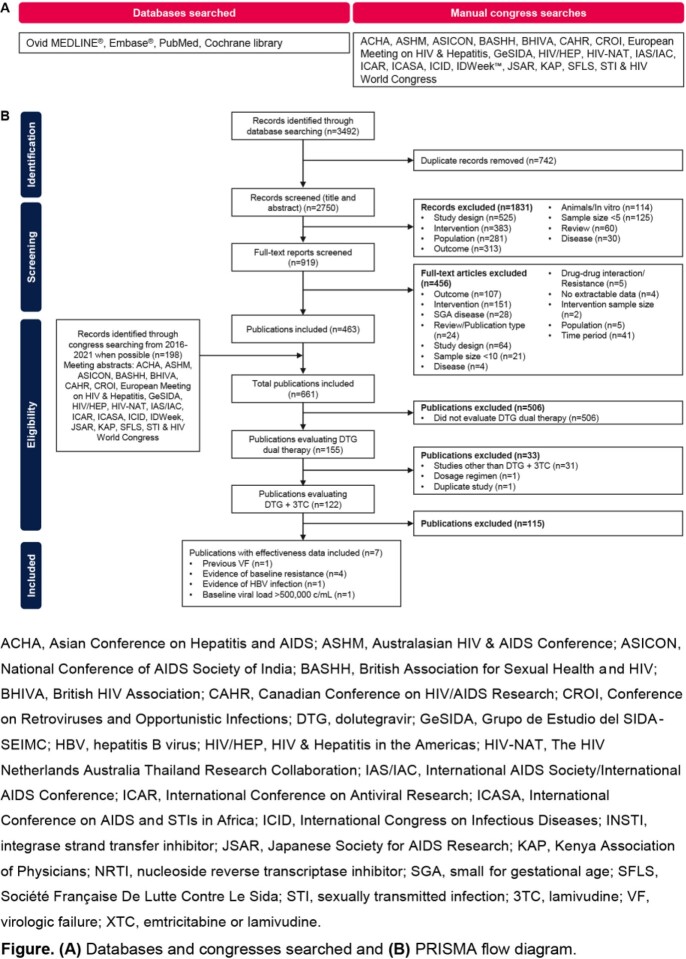

**Figure:**
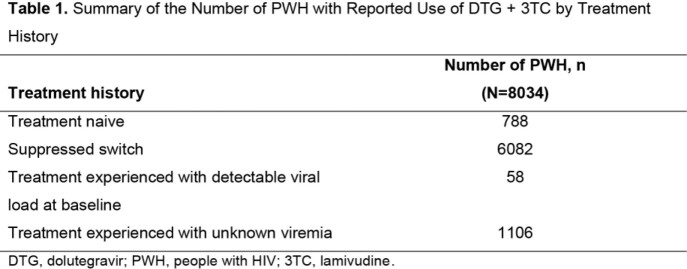

**Figure:**
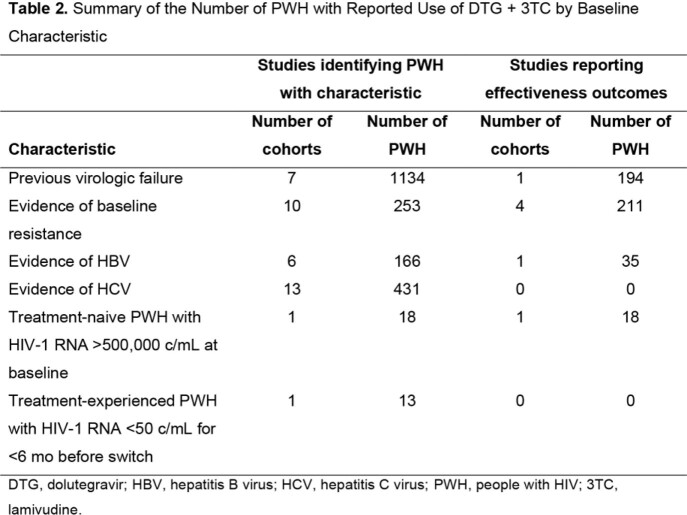

**Conclusion:**

DTG + 3TC has been used in PWH with various baseline characteristics, including RCT exclusion criteria. Outcomes from published RWE in these subgroups further support the clinical data demonstrating the high effectiveness and barrier to resistance of DTG + 3TC.

**Disclosures:**

**Jihad Slim, MD, FACP**, abbvie- speaker bureau: Honoraria|Gilead Speaker Bureau: Honoraria|Janssen Speaker Bureau: Honoraria|Merck Speaker Bureau: Honoraria **Douglas Ward, MD**, ViiV Healthcare: Advisor/Consultant|ViiV Healthcare: Honoraria **Stefan Schneider, MD**, GlaxoSmithKline: Grant/Research Support|Janssen: Grant/Research Support|ViiV Healthcare: Advisor/Consultant|ViiV Healthcare: Grant/Research Support **Madhusudan Kabra, BPharm, MSc**, GlaxoSmithKline: Stocks/Bonds|ViiV Healthcare: Employee **Gustavo Verdier, BSc, BPharm, MBA**, GlaxoSmithKline: Stocks/Bonds|ViiV Healthcare ULC: Salary **Clifford B. Jones, BSc MSc MB ChB**, GSK: Stocks/Bonds|viiv healthcare: Employee **Emilio Letang, MD, MPH, PhD**, GlaxoSmithKline: Stocks/Bonds|ViiV Healthcare: Employee.

